# The SOS-SAH questionnaire in clinical practice: a multi-method evaluation study

**DOI:** 10.1186/s12883-023-03280-w

**Published:** 2023-06-19

**Authors:** E. Nobels-Janssen, I. L. Abma, I. R. de Ridder, R. H. L. Haeren, M. H. Hertog, D. Nanda, B. van der Pol, W. I. M. Verhagen, R. H. M. A. Bartels, P. J. van der Wees, H. D. Boogaarts

**Affiliations:** 1 Department of Neurology, Canisius Wilhelmina Hospital, Nijmegen, the Netherlands; 2 Department of Neurosurgery, Radboud University Medical Center, Nijmegen, the Netherlands; 3 IQ healthcare, Radboud University Medical Center, Radboud Institute of Health Sciences, Nijmegen, the Netherlands; 4 Department of Neurology, Maastricht University Medical Center, Cardiovascular Research Institute Maastricht, Maastricht, the Netherlands; 5 Department of Neurosurgery, Maastricht University Medical Center, Maastricht, the Netherlands; 6 Department of Neurology, Isala Hospital, Zwolle, the Netherlands; 7 Department of Neurosurgery, Isala Hospital, Zwolle, the Netherlands; 8 Department of Neurosurgery, Elisabeth-TweeSteden Hospital, Tilburg, the Netherlands

**Keywords:** Patient-reported outcome measure, Aneurysmal subarachnoid hemorrhage, SOS-SAH

## Abstract

**Background:**

In patients with mild disabilities after aneurysmal subarachnoid hemorrhage (aSAH), invisible symptoms might be easily overlooked during consultations in the outpatient clinic. We hypothesize that the Questionnaire for the Screening of Symptoms in aneurysmal Subarachnoid Hemorrhage (SOS-SAH), a disease-specific patient-reported outcome measure, might aid in screening for symptoms after aSAH. The objective of this explorative study is to evaluate the perceived impact of using the SOS-SAH in daily clinical practice for patients after aSAH, as well as to explore potential barriers to further implementation.

**Methods:**

This multi-method study consists of a quantitative and a qualitative component. To evaluate differences in quality of care, a patient experience survey was sent to patients receiving usual care and to patients who received the SOS-SAH. A multiple linear regression model was applied, with the intervention group and case mix adjusters as independent variables. We described differences in the number of symptoms discussed between patients receiving usual care and those receiving care post-implementation. Following implementation, 16 patients and 6 healthcare professionals were interviewed about their perceptions concerning the impact of and barriers to using the SOS-SAH. A thematic analysis was performed to identify the main themes.

**Results:**

The survey did not reveal any differences between the usual-care group and the post-implementation group on the scales of the patient experience survey. After implementation of the SOS-SAH, the number of symptoms discussed during consultation did not increase. The interviews suggest that the SOS-SAH may improve the preparation of patients by providing them with greater insight into their complaints and by raising issues for the consultation. It could also enhance the structure and efficiency of consultation, in addition to improving communication about issues that matter to patients. All patients and healthcare professionals recommended continuing the use of the SOS-SAH in daily practice.

**Conclusions:**

Although no quantitative improvements were found in patient experience and symptoms discussed during consultation, implementation of the SOS-SAH could aid in screening for symptoms in patients after aSAH, and it might have a positive influence on patient preparation, while helping to structure consultations between patients and healthcare professionals.

**Supplementary Information:**

The online version contains supplementary material available at 10.1186/s12883-023-03280-w.

## Introduction

Patient-reported outcome measures (PROMs) are questionnaires that reflect the views of patients with regard to their own health, and are nowadays often used for improving patient-centered healthcare [1]. By offering a patient’s perspective on symptoms, PROMs are important, given that most healthcare interventions are aimed at reducing the burden of disease, and regular assessments may overlook some symptoms. The use of PROMs can empower patients to arrive at shared decision-making. They could also be applied as an instrument for monitoring symptoms over time or for evaluating the effects of treatment [2].

This study focuses on the use of a PROM in patients after an aneurysmal subarachnoid hemorrhage (aSAH), which is caused by the rupture of an intracranial aneurysm and that results in the accumulation of blood in the subarachnoid space. Even after successful treatment, half of all survivors suffer from debilitating symptoms, including cognitive problems, fatigue, mood disorders, speech disturbances, or disabilities in self-care [3–6]. These symptoms are nevertheless easily overlooked during follow-up visits to the outpatient clinic [7]. Healthcare professionals (e.g., neurosurgeons, neurologists, intervention radiologists, specialized nurses) tend to focus primarily on the consequences of the more prominent and visible signs and symptoms, in addition to discussing the result of additional investigations and potential upcoming treatments.

To address the issues outlined above, we recently developed the Questionnaire for the Screening of Symptoms in aneurysmal Subarachnoid Hemorrhage (SOS-SAH) to screen for symptoms in patients with mild disabilities after aSAH, to facilitate the identification of symptoms that often remain undetected [7]. The SOS-SAH was developed primarily using domains and items from existing PROMs, and it consists of 14 domains: cognitive abilities, hypersensitivity to stimuli, anxiety, depression, fatigue, social roles, personality change, language, vision, taste, smell, hearing, headache, and sexual function. It also includes a proxy measurement for use by family members to assess cognitive functioning and personality changes (Supplemental Table 1).

We aimed to explore the perceived impact of the SOS-SAH in the follow-up care of patients after aSAH, as well as potential barriers to and facilitators of further implementation. We hypothesized that the SOS-SAH:

- encourages patients to reconsider the symptoms and/or limitations they experience after the aSAH.
- aids patients in preparing for their follow-up appointments with their healthcare professionals.
- improves the quality of care experienced by patients.
- gives healthcare professionals a quick, comprehensive overview of the patient’s symptoms, thus possibly enhancing the efficiency of the consultation and improving the explanation of complaints experienced by patients.

A combination of quantitative and qualitative research methods was used, in order to provide a full overview of the impact of the SOS-SAH.

## Methods

### Study design

**Fig. 1:**
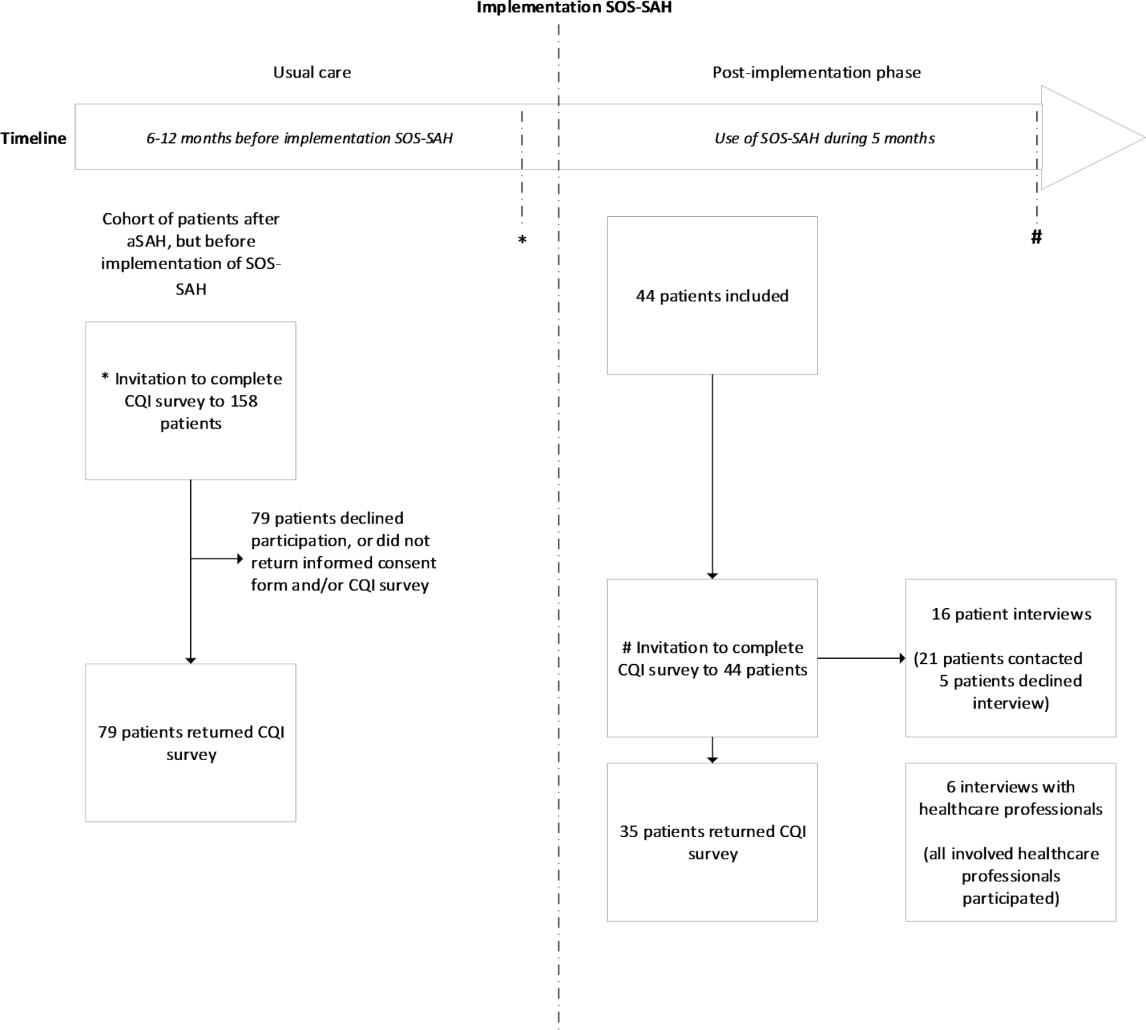
Participant study flow and data collection

In this multi-method, multicenter explorative study, the SOS-SAH was implemented in the clinical practice of four hospitals in the Netherlands. A multi-method design (quantitative surveys and qualitative interviews) was chosen to evaluate both the instrument’s impact on perceived quality of care in general and to provide insight into the perspectives and experiences of patients and healthcare professionals with the SOS-SAH. We conducted a survey among a group of patients receiving usual care, as well as in a group receiving care after the implementation of the SOS-SAH. We further conducted qualitative interviews with patients in the post-implementation group and healthcare professionals. The various methods are described in more detail in the following sections and summarized in Fig. 1. Ethical approval was not required for this type of study under Dutch law, and an exemption was obtained by the local Medical Ethics Committee (CMO region Arnhem-Nijmegen, file number 2018–4937). All patients provided written informed consent.

### Implementation of the SOS-SAH

A paper version of the SOS-SAH was sent to patients two weeks prior to their follow-up hospital appointment. They were asked to complete the SOS-SAH at home and bring the completed questionnaire to their appointment. After checking in for the appointment, their answers were entered in a digital system (Quality Registry NeuroSurgery), and a summary report was generated (Supplemental Fig. 1), with a green, orange, or red smiley indicating the level of problems experienced by the patient in each domain. This summary report was presented to the healthcare professional. All healthcare professionals were informed by the principal investigator (EN) about the intention of using the SOS-SAH and different options for the implementation of the SOS-SAH were discussed. The healthcare professionals in each center were encouraged to implement the SOS-SAH within their own workflow in the way they thought to be most convenient and an implementation plan was discussed. In three hospitals the involved nurse practitioners used the SOS-SAH and in the other hospital the involved physicians. This has resulted in two hospitals in which one healthcare professional used the SOS-SAH and in the other hospitals two healthcare professionals used the SOS-SAH.

### Setting and participants

Between January 2021 and January 2022, consecutive patients were included in this study during their first admission for aSAH. Four hospitals participated: two academic medical centers and two general hospitals. In each hospital, all eligible patients were approached for inclusion during a five-month period. Patients were eligible if they had a recent diagnosis of aSAH, were ≥ 18 years of age, and were scheduled for a follow-up consultation in the participating hospital after their initial management. Patients who were not fluent in Dutch or were illiterate and who had nobody to help them complete the questionnaire, or were otherwise not able to complete the questionnaire were excluded.

Prior to the implementation of the SOS-SAH, a usual-care group of 40 consecutive patients per center who had survived aSAH 6–12 months before the study, were invited to complete a survey about their experienced quality of care. The modified Rankin Scale (mRS) score after six months and the World Federation of Neurosurgery Score (WFNS) at admission were obtained from medical records.

A selection of patients who had completed the SOS-SAH were invited for an interview after their consultation in the hospital (when the SOS-SAH was used). For practical reasons, in two hospitals, we selected patients who had used the SOS-SAH most recently. In the other two hospitals, we selected patients in chronological order of inclusion (except for the first patient, due to potential start-up problems in the implementation of the SOS-SAH). Patients were included if they could be reached by telephone and were willing to participate. We interviewed 16 out of 21 invited patients, either in person (n = 6) or by video call (n = 10) (Fig. 1). After 16 interviews, no new topics were identified, and we concluded that data saturation had been reached.

Each of the healthcare professionals participating in the study were invited for an interview to evaluate the impact of the use of the SOS-SAH on clinical practice. The interviews were scheduled after the end of the five-month study period in their hospitals.

Participation was voluntary, and data were handled anonymously. All patients and healthcare professionals provided written informed consent.

### Patient experience survey

A survey was used to compare the quality of care experienced by patients who did not receive the SOS-SAH and patients who did receive the SOS-SAH. We used an adapted version of the Consumer Quality Index (CQI) for patients after ischemic or hemorrhagic stroke (CQI-CVA). The CQI is a standardized, validated patient experience survey developed by the Netherlands Institute for Health Services Research [8, 9]. The CQI-CVA consists of 79 items and eleven scales about patients’ experiences with quality of care in various healthcare institutions (e.g., hospital, rehabilitation center, paramedical therapy). The response options for some items were adjusted to make the survey more suitable for patients after aSAH (e.g., the question on which healthcare professional had performed the follow-up). Most questions were scored on a four-point Likert scale. Each of the eleven scales is scored separately and can be interpreted separately from the other scales. To keep the survey brief, we used only the four scales from the survey that we considered most relevant, with two, three, or four questions per scale (total 12 items). These scales covered: follow-up care in the outpatient clinic, collaboration between healthcare professionals, information about support options (e.g., patient associations or the availability of a designated contact), and support in gaining access to healthcare or medical devices (Supplemental Table 2). The questions about age, sex, educational level, and experienced quality of health, were used as case-mix adjusters and to describe the characteristics of the patients in the different groups. Furthermore, one question was added to evaluate whether the SOS-SAH had improved the detection and discussion of symptoms (Question 31, Supplemental Table 2). Patients could indicate whether they had experienced each of a series of symptoms and whether they had discussed these symptoms with their healthcare professionals.

### Interviews

Semi-structured interviews performed by a trained interviewer (EN) were conducted with patients and healthcare professionals. The eliciting questions were broad, allowing participants to raise the experiences they considered most relevant. Prompts were used to allow patients and healthcare professionals to elaborate on their views and experiences with the SOS-SAH (for additional information, see Supplemental material I). The main goal of the interview with patients was to assess how they had experienced completing the SOS-SAH and to gather their perceptions concerning the impact of the SOS-SAH on the consultation and their preparation for it. The main topics of the interview with healthcare professionals were experiences with the SOS-SAH, perceived advantages and disadvantages of the instrument, its impact on the care provided, and any potential barriers to or facilitators of the implementation of the SOS-SAH.

### Data analysis

Analysis was performed using SPSS version 25. Missing data were deleted pairwise. Descriptive statistics were used to summarize the characteristics of the patients.

### Quantitative analysis

Scale scores on the CQI were valid only if the patient had answered at least 50% of the questions within a scale. For the computation of scale scores, items consisting of only two response options (e.g., yes/no) were recoded. Items with negative wording were reversed. To test for internal consistency within each scale, the Cronbach’s alpha score was calculated. A Cronbach’s alpha score of > 0.70 was considered good, with scores of 0.60–0.70 regarded as acceptable [8]. This was done to evaluate whether these items also belonged to one of the scales in our study sample of patients after aSAH. Only patients who actually used the SOS-SAH were included in the following analyses to measure any differences in quality of care due to the implementation of the SOS-SAH. The intraclass correlation coefficient for center, using the scale of the CQI survey as a dependent variable, was close to zero, indicating that the use of a linear mixed model was not necessary. To correct for case-mix adjusters, a multiple linear regression model was applied for each of the four CQI scales separately, with the CQI scale scores as dependent variables and with intervention group (usual care or implementation), as well as age, sex, educational level, mRS, self-reported health, and WFNS as independent variables. We described differences in the number of symptoms discussed between patients receiving usual care and those receiving care after the implementation of the SOS-SAH, based on responses to Question 31 of the survey (Supplemental Table 2).

### Qualitative analysis

All interviews were audio-taped and transcribed verbatim. Thematic analysis was performed in ATLAS.ti 9.1.6, using open coding followed by axial coding (i.e., making connections between codes to organize them into new categories), in order to identify main themes that best described the experiences and views of patients and healthcare professionals with regard to the SOS-SAH. EN and IA independently coded five interviews and reached consensus through discussion on a preliminary version of the code book. Thereafter, EN coded all remaining interviews, adding new codes as needed. IA critically checked all interviews and new codes for agreement. EN searched for relationships between codes, and themes and subthemes were derived from the data. EN, IA, HB, and PW held a collaborative meeting to discuss and reach consensus on the codes, themes, and subthemes. The code book and coding were revised as needed.

## Results

An overview of the patients included is presented in Table 1. In all, 79 of the 158 (50%) patients in the usual-care group completed the CQI survey (Table 2). A total of 44 patients were included to use the SOS-SAH. Most of these patients (38; 86%) completed the SOS-SAH, and 35 (79%) also completed the CQI survey. The WFNS scores of patients in the post-implementation group were lower (i.e. representing better scores) than those of patients in the usual-care group (Table 1).

**Table 1:** Patient characteristics

		Usual-care group		Post-implementation group			p-value^#^
		Total (n = 158)	Responders (n = 79)	Total (n = 44)	Patients that completed the SOS-SAH and CQI survey (n = 34)	Patients interviewed (n = 16)	
Age		58.0^1^ (11.8)	59.0^1^ (10.8)	57.0^1^ (10.5)	57.4^1^ (10.5)	53.5^1^ (11.2)	0.288
Sex							0.286
	Male	51 (32%)	25 (32%)	18 (41%)	16 (47%)	10 (63%)	
	Female	107 (68)	54 (68%)	26 (59%)	18 (53%)	6 (38%)	
WFNS grade							**0.030**
	I	81 (51%)	37 (47%)	33 (75%)	26 (77%)	14 (88%)	
	II	42 (27%)	25 (32%)	7 (16%)	5 (15%)	1 (6%)	
	III	2 (1%)	2 (3%)	1 (2%)	1 (3%)	0	
	IV	19 (12%)	9 (11%)	1 (2%)	1 (3%)	1 (6%)	
	V	14 (9%)	6 (8%)	1 (2%)	0	0	
	Missing	0	0	1 (2%)	1 (3%)	0	
mRS							0.872
	0	20 (13%)	13 (17%)	5 (11%)	4 (12%)	3 (19%)	
	1	54 (34%)	26 (33%)	14 (32%)	10 (29%)	5 (31%)	
	2	44 (28%)	25 (32%)	10 (23%)	8 (24%)	5 (31%)	
	3	16 (10%)	6 (8%)	2 (5%)	2 (6%)	0	
	4	9 (6%)	5 (6%)	1 (2%)	0	0	
	5	2 (1%)	0	0	0	0	
	Missing	13 (8%)	4 (5%)	12 (27%)	10 (29%)	3 (19%)	
Center							0.652
	1	40 (25%)	23 (29%)	14 (32%)	11 (32%)	5 (31%)	
	2	38 (24%)	20 (25%)	8 (18%)	7 (21%)	4 (25%)	
	3	40 (25%)	19 (24%)	13 (30%)	8 (24%)	4 (25%)	
	4	40 (25%)	17 (22%)	9 (20%)	8 (24%)	3 (19%)	
Educational level							
	No degree		1 (1%)		0	0	
	Basic preparatory secondary vocational education		3 (4%)		2 (6%)	1 (6%)	
	Preparatory secondary vocational education		17 (22%)		8 (24%)	3 (19%)	
	Intermediate general secondary education		16 (20%)		6 (18%)	1 (6%)	
	Senior secondary vocational training		19 (24%)		7 (21%)	3 (19%)	
	Senior secondary general education/pre-university education		3 (4%)		3 (9%)	1 (6%)	
	Higher professional education		14 (18%)		6 (18%)	4 (25%)	
	Academic higher education		4 (5%)		1 (3%)	0	
	Missing		2 (3%)		1 (3%)	3 (19%)	
Experienced quality of health							
	Excellent		2 (3%)		2 (6%)	2 (13%)	
	Very good		8 (10%)		7 (21%)	4 (25%)	
	Good		45 (57%)		19 (56%)	7 (44%)	
	Fair		20 (25%)		3 (9%)	1 (6%)	
	Poor		1 (1%)		2 (6%)		
	Missing		3 (4%)		1 (3%)	2 (13%)	

Percentages are rounded to the nearest whole number and thus totals may not add to exactly 100%

^1^ Mean (standard deviation)

^#^ p-values are presented for the total usual care group compared to the total implementation group, using a T-test for age and a Chi-Square test for sex, WFNS, mRS, and center

Abbreviations: mRS: modified Rankin Scale; WFNS: World Federation of Neurosurgical Societies

**Table 2:** Response percentages for CQI survey

	Usual care total (n = 158)	Implementation: patients that completed SOS-SAH (n = 38)	p-value
Completed CQI	79 (50%)	34 (89%)	
Time between aSAH and CQI completion (days)	235^1^ (106–666)	167^1^ (95–337)	**0.000**

^1^ Median (range)

### Survey results

In general, patients in the post-implementation group had fewer symptoms than patients in the usual-care group, although the proportion of symptoms that were not discussed during the consultation relative to those that were discussed is the same for both groups (a ratio of 0.35 for usual-care, a ratio of 0.31 for post-implementation) (Supplemental Table 3). The internal consistency of the scales of the CQI was acceptable to good, with the following Cronbach’s alpha scores for the scales: follow-up care in the outpatient clinic 0.82, collaboration between healthcare professionals 0.81, information about support options 0.62, and support in gaining access to healthcare or medical devices 0.65. Using multiple linear regression models, we found no significant difference between the usual-care and the post-implementation group for any of the CQI scales (Table 3).

**Table 3:** Multiple linear regression results for all four scales of the CQI survey used as the dependent variable

Scales of the CQI survey	Independent variable	R [2]	B-value (95% CI)	p-value
Follow-up care in the outpatient clinic	Group (usual-care or post-implementation)	0.181	0.083 (-0.402–0.567)	0.735
Collaboration between healthcare professionals	Group (usual-care or post-implementation)	0.321	0.279 (-0.116–0.674)	0.162
Information about support options	Group (usual-care or post-implementation)	0.125	-0.071 (-0.555–0.414)	0.773
Support in gaining access to healthcare or medical devices	Group (usual-care or post-implementation)	0.243	0.592 (-0.056–1.239)	0.073

These analyses were corrected for the variables: age, sex, mRS, WFNS, educational level, and self-reported health

### Interviews

Interviews were conducted with 16 patients and 6 healthcare professionals: 1 neurologist, 1 neurosurgeon, 1 physician assistant, and 3 specialized nurses.

### Use of the SOS-SAH during consultation

In general, the healthcare professionals used the SOS-SAH in their consultations in the same way. They started the consultation with an open-ended question to ask how the patient was doing, after which they discussed the results of the SOS-SAH, guided by the summary report with smileys, and focusing on the domains with the lowest scores. One of the healthcare professionals did not have the summary report available before the consultation, and used the completed questionnaire itself to discuss potential complaints.

### General opinion on the SOS-SAH

Although all patients recognized the potential value of the SOS-SAH, not all of them reported that the use of the PROM had made a positive impact on their care, largely because some patients had not experienced any residual complaints after their aSAH. All participating healthcare professionals noted that the SOS-SAH consists of relevant items, that it is comprehensive, and that it provides a quick and accurate overview of the problems.

### Impact of the SOS-SAH

We identified three main themes regarding the potential impact of the SOS-SAH. These three main themes and the identified subthemes are presented in Table 4 and are described in detail below.

**Table 4:** Themes and subthemes identified with regard to the impact of the SOS-SAH.

Impact of the SOS-SAH				
**Themes**	**Subthemes**	**Topics of the underlying codes**	**Mentioned by patients**	**Mentioned by healthcare professionals**
Impact on preparing for the consultation	Awareness	Patient awareness of complaints makes discussion easier	X	X
		Opportunity for patients to compare their own complaints with those of others	X	
		Insight into possible improvement in complaints	X	X
	Emotions of patients	Reassuring	X	X
		Confrontational	X	X
		Patients feel heard	X	X
		Wrong color of smiley can lead to frustration		X
	Greater involvement of family members	Proxy questions triggered conversation	X	X
Impact on consultation	Easy overview	Good overview	X	X
		Insight into complaints	X	X
		Insight into possible complaints		X
	Arriving at the most important topics more quickly	Saves time		X
		Efficient consultation		X
		Focus on domains with the lowest scores	X	X
		Risk of omitting certain domains		X
	Improved structure	Provides patients with insight into the course of consultation		X
		Helps to maintain focus when emotions take over		X
		Check whether all complaints are discussed		X
	Discussion of topics	More topics for discussion, especially sexuality and personality	X	X
		Patients share their concerns more easily		X
		Proxy questions make it possible to confront patients		X
	More and focused explanation	Identifying important problems allows the opportunity to discuss them		X
	Greater role of family members	Greater involvement of family members		X
Impact on follow-up actions	Targeted follow-up actions	Used in letter of referral to rehabilitation physician		X
		Impact dependent on rehabilitation care received		X

### Impact on preparing for the consultation

*Awareness –* The majority of patients indicated that completing the SOS-SAH generated greater insight and awareness regarding their health complaints, which helped them in preparing for their consultation (Table 5, Quote 1). They expressed that they were better able to discuss their complaints and to formulate questions. Healthcare professionals confirmed that patients seemed better prepared for the consultation after completing the SOS-SAH (Table 6, Quote 1). Some patients also indicated that the SOS-SAH provides insight into which complaints other patients generally experience. As noted by some patients, however, uncertainty can arise if a complaint that they have experienced is not included on the questionnaire, leaving them to wonder whether other patients might not experience the same complaint. Both patients and healthcare professionals appreciated the fact that repeated measurements could also offer insight into improvements in complaints.

*Emotions –* Patients and healthcare professionals indicated that the questionnaire can reassure patients (e.g., about the number of complaints or as an illustration what is actually going well). In addition, patients noted that being asked to complete the SOS-SAH reflected interest in them, and healthcare professionals observed that it might lead to patients feel as if they are being heard. Both healthcare professionals and patients identified that the questionnaire could also be perceived as confrontational, however, causing patients to re-experience emotions relating to their aSAH and hospital stay. Furthermore, one healthcare professional mentions the risk that patients might feel frustrated if they do not think that the color of the smileys in the summary report represents the actual severity of their complaints.

*Greater involvement of family members –* For some patients, the proxy questions served as a catalyst for conversation between them and their family members about the actual situation and complaints. This was generally appreciated.

### Impact on consultation

*Easy overview –* Patients and healthcare professionals indicated that the SOS-SAH provided good insight into a patient’s complaints, both for themselves and for their healthcare professionals.Furthermore, healthcare professionals said that the SOS-SAH reminded them of many invisible complaints that patients with aSAH might experience which stimulated the professionals to discuss these topics.

*Arriving at the most important topics more quickly –* Healthcare professionals indicated that the SOS-SAH helped them to get to the core of a patient’s problems more quickly (Table 6, Quotes 2 and 3), thus leading to an efficient consultation. They suggested this can save time, which can then be used to provide more explanation to the patient. As noted by some healthcare professionals, however, domains with low scores may still be important to discuss with the patient and not referring to them could therefore result in not discussing complaints that actually do matter to patients.

*Improved structure –* Healthcare professionals noticed that the SOS-SAH assigns structure to consultations in multiple ways (Table 6, Quote 4). First, when patients become emotional, it can help to maintain a focus on the main topics. Second, it can provide patients with insight into the course of the consultation. Third, it offers healthcare professionals the opportunity to check whether all complaints have been addressed.

*Discussion of topics –* Healthcare professionals mentioned that they discussed more and sometimes different topics than they usually do, especially with regard to sexuality and personality changes. The majority of patients indicated that the SOS-SAH had helped them formulate new questions to address in their consultation (Table 5, Quote 2). A patient indicated that the use of the SOS-SAH may also lead patients to ask fewer questions, however, if they feel that answering the questionnaire eliminates the need for any further discussion.

*Greater role of family members –* The proxy questions also offered the opportunity to confront patients with any differences between their own views and those of their proxies and to explore these differences.

*More and focused explanation –* Some healthcare professionals indicated that SOS-SAH improved their focus on the problems of that a patient is experiencing, therefore offering an opportunity to provide additional explanation about these problems.

### Impact on follow-up actions

Most healthcare professionals indicated that they had not adjusted the treatment or referral of patients based on the SOS-SAH. Many patients had already received rehabilitative care, and no adjustments were needed. At the same time, however, some healthcare professionals had used the SOS-SAH to inform rehabilitation physicians about the problems a patient had experienced in the referral letter (Table 6, Quote 5).

**Table 5:** Quotes from patients

Impact on preparation	
1) “I think, for me, it mostly led to a bit of awareness, because, yeah, you have had something really serious. (…) As time passes, you don’t think that you’ve had anything else. And this list lets you describe [what you have experienced] and also the different aspects of it, which for me, yeah, are especially related to thinking and that sort of thing. Yeah, you also read a lot of things that are fortunately not applicable, like physical problems. Well, you see, it gives you a sort of recognition when you read it. Mood swings, fatigue, concentration, those kinds of things, yeah, that does make you aware. And if you can indicate what that does to you, it’s a good thing, right, that you can start a conversation, that you’ve already been able to think about it.” [Patient 1, Center 2].	
Impact on consultation	
2) “…but it also gave us starting points to talk about how things are going now, because what still bothers me is that I’m tired. Well, yeah, we talked about that, what causes it, and what the expectations are.” [Patient 8, Center 1].	
Practical considerations	
3) “‘How spent do you feel on average?’ I thought that was a strange word in the questionnaire, you know, and then it says ‘How tired were you on average?’ Yeah, spent and tired, they fall into the same category for me, and then I think, ‘What do they want,’ and, in purely linguistical terms, I found ‘spent’ a strange word for a questionnaire. (…) Right, and you know what it is, ‘spent,’ the word ‘spent,’ the word ‘spent’ inherently implies that you’re at the end of the scale; you can’t be a little bit spent.” [Patient 4, Center 3]	
4) [regarding Question 2 of the SOS-SAH: “I have been able to focus my attention”] “But that really depends on the setting, so if I close myself off, I can focus well. And if there’s a lot going on around me, then I can only do one thing at a time.” [Patient 15, Center 4]	
5) Interviewer: “And you said at the beginning, ‘I also have problems with [the Dutch organization that issues driver’s licenses] and with quitting smoking.’ You said, ‘I [didn’t consider those things when answering] the questionnaire.’”. Respondent: “Yes, I tried to. (…) I don’t know exactly off the top of my head [what influence it had], but, for example ‘Are you worried?’ I am worried for example, about whether I’ll have my driver’s license again in the foreseeable future. I can laugh and see things on the bright side. I can do that, and I can do that just as much as I used to, but I’m still worried about my driver’s license, and those worries also make it harder for me to quit smoking, everything influences everything else.” [Patient 7, Center 1]	
6) “It [the SOS-SAH] asks about the past 7 days. I also indicated that I was on vacation, so that’s a different story than the week before. I think I also got different results.” [Patient10, Center 1]	

**Table 6:** Quotes from healthcare professionals

Impact on preparation	
1) “I don’t know whether [patients] mention [their complaints] more frequently, but I do think they can formulate it better, because a questionnaire has already been completed beforehand, which actually made them think about, oh yeah, the feeling that things aren’t going well at home, or that things are actually going well and what is causing that, and where any problems might be.” [Healthcare professional 3, Center 1]	
Impact on consultation	
2) “And now you already have a bit of preparation, which, yeah, I think helps you, in the already limited time, to be quicker in getting around to topics that actually do matter to [patients].” [Healthcare professional 1, Center 2]	
3) Interviewer: “So you say that it can change healthcare. But how exactly would it change healthcare, such a questionnaire?”	
Healthcare professional: “Yes, so I think, more personalized medicine. So that you get more specific, you know, I could do this with this patient—I should ask about it. I should focus on that.” [Healthcare professional 5, Center 4]	
4) “I think that, more than anything, it’s a very nice, structured way for both patients and myself to use it as a frame for the consultation, because it quickly gives you an idea of what you can ask the patients or family members to explain. So, that’s the biggest gain for me.” [Healthcare professional 6, Center 4]	
Impact on follow-up actions	
5) “If we noticed that [the current care was not sufficient], then I used [the SOS-SAH] in the referral to the rehabilitation physician as a brief summary of what the complaints were and what the patient was encountering in daily life at home, and I requested [the rehabilitation physician] to invite the patient for a consultation.” [Healthcare professional 3, Center 1]	
Practical considerations	
6) “Then [patients] mention something or they indicate that they want to share something with you, but what it [the SOS-SAH] says is not conclusive. It’s, yeah, I think it’s very difficult for such a questionnaire to summarize how people really feel.” [Healthcare professional 2, Center 1]	

### Practical considerations

We identified two practical considerations to consider before implementing the SOS-SAH, which are outlined below and in Table 7.

**Table 7:** Practical considerations with regard to the implementation of the SOS-SAH.

Practical considerations before implementation of the SOS-SAH				
**Themes**	**Subthemes**	**Topics of the underlying codes**		
Practical considerations	Validity	Problems with completing SOS-SAH	X	
		Discrepancies between complaints and smileys	X	X
		Influences of other factors	X	X
		Initial patient situation important to interpretation of the SOS-SAH	X	
		Last 7 days can give an inaccurate representation of complaints	X	
	Logistical difficulties	Forgot to complete or discuss the SOS-SAH	X	X
		Logistical issues for use in consultation		X
		Digital completion not always possible after SAH	X	X
		Incorporate questionnaire into electronic patient record		X

*Validity –* Half of the patients experienced problems completing the questionnaire. These problems included the following: (1) difficulty pointing out the difference between their current complaints compared to before the aSAH; (2) illogical linguistic formulation of a question (Table 5, Quote 3); and (3) difficulty indicating the extent of limitation if a patient has not yet resumed an activity (e.g., work). Patients mentioned that the way in which they answered the questions might have been influenced by the initial situation before the aSAH, a patient’s character before the aSAH, the time between aSAH and completion of the SOS-SAH, and the activities of the past week (Table 5, Quotes 4, 5, 6). For example, one patient mentioned the influence of having quit smoking on anxiety, and another referred to the impact of losing a driving license on work activities. One healthcare professional also recognized that the SOS-SAH might not provide an accurate representation of how a patient is functioning and that it is important to discuss the questionnaire with the patient (Table 6, Quote 5).

All healthcare professionals regarded the summary report with smileys as providing a realistic presentation of the complaints of patients. Some nevertheless noted that there were occasional discrepancies between the complaints and the smileys presented on the report, with the smileys either under-representing or over-representing the actual severity of complaints. None of the healthcare professionals regarded this as a barrier to the use of the SOS-SAH, as discrepancies were revealed and discussed during the consultation.

*Logistical difficulties –* In some cases, patients, proxies, and healthcare professionals forgot to use the SOS-SAH. Furthermore, healthcare professionals reported that collecting the SOS-SAH data in daily practice requires time and effort. Although the logistical process was often arranged by a research nurse during this study, some healthcare professionals mentioned that, in ordinary daily practice, the necessary rearrangement of processes within the outpatient clinic would likely pose a barrier to using the instrument. Healthcare professionals recommended incorporating the SOS-SAH into the electronic patient records. Some healthcare professionals suggested that a digital questionnaire might improve the logistical process, and they were confident that patients would be capable of completing the SOS-SAH digitally. In contrast, half of the patients interviewed expressed a preference for completing the questionnaire on paper or indicated that they would not able to complete it digitally.

## Discussion

This multi-method explorative study identified several possible advantages of implementing the SOS-SAH in clinical practice in follow-up care for patients with aSAH. The survey results did not provide any evidence that implementation of the SOS-SAH improved experienced quality of care, as measured on the CQI survey. Also, the proportion of symptoms discussed during consultations did not change after implementation of the SOS-SAH. From the interviews, it became clear that the SOS-SAH can improve a patient’s preparation by providing greater insight into actual or potential problems, as well as by posing questions or raising issues for the consultation. In addition, healthcare professionals noted that the SOS-SAH improved the structure and efficiency of consultations and improved communication about issues that matter to patients. All healthcare professionals saw advantages to the SOS-SAH and recommended continuing the use of the instrument in daily practice. In general, healthcare professionals had a more positive view towards the SOS-SAH than patients did, although none expressed a negative opinion about it.

There was no difference between the usual-care and post-implementation groups with regard to quality of care, as measured by the CQI survey. We have identified several reasons for the lack of such difference. First, the CQI survey is not validated for use in patients after aSAH, although we did adapt it for use in this patient group. We nevertheless did not assess content validity, and the relevance of the items for patients after aSAH was an assumption of the research team. Second, previous studies have indicated that the discriminant validity of the CQI-CVA for measuring differences in quality of care between centers is limited [8]. Third, the timing of the distribution of the CQI survey (in relation to the aSAH) differed for the patients receiving usual care and those seen after implementation of the SOS-SAH (the last having a shorter time between the survey and aSAH). This difference in timing might have influenced the patients’ views on the quality of care. It could be that some symptoms would be discussed during subsequent consultations that had not yet taken place in the post-implementation group. Fourth, due to the small size of the post-implementation group and the difference in patient characteristics between the two groups, the results might not provide a reliable measure of differences in the quality of care. In fact, patients in the post-implementation group have a better WFNS score at admission, suggesting that they might have a better outcome, although this is not confirmed by the mRS. Finally, it is also important to mention that quality of care is a concept that is difficult to measure and that cannot be fully understood without considering social norms and values [10]. Measurements of the quality of care reveal only subtle differences between patients, and there is little variation among answers [11, 12].

The use of PROMs might have a positive effect on the process of care, including patient-clinician communication [2, 13]. Two theoretical ways have been proposed for how the use of such indicators could support clinician-patient communication and patient care. First, the completion of PROM instruments helps patients to raise issues with their clinicians. Second, PROM scores can raise clinicians’ awareness of the problems experienced by their patients [14]. This suggests that the process of completing PROM instruments is not simply a way to retrieve information from patients, but that it can change how patients think about their conditions [14]. Our findings support these theories.

Information obtained from the interviews identifies two practical considerations regarding the application of the SOS-SAH: the validity of the questionnaire and logistical difficulties in its implementation. These considerations are in line with previously identified challenges in the use of PROMs [1]. In the development of the SOS-SAH, we considered how the validity of the questionnaire could be optimized, and we have developed several recommendations for overcoming some of the practical considerations identified in this study [7]. First, the validity of the questionnaire is enhanced by the fact that it is composed primarily of existing and previously validated questionnaires. The results could be influenced by complaints inherent to aSAH (e.g., cognitive symptoms and a decrease in patients’ insight into their own performance due to brain damage). For this reason, we added proxy questions on cognitive functioning and personality change. In addition, the results of the SOS-SAH must be discussed in order to make sense of the scores. Previous studies have also indicated that PROMs might not be capable of fully capturing the complexity or dynamic nature of patients’ symptoms [14]. Some of the patients interviewed in this study suggested that the questionnaire should be more individualized, customized to the level of complaints occurring during admission. It is important to note, however, that the standardization required to support psychometric validity of the PROM does restrict sense-making for questions that are not applicable to the specific situations of individual patients [14] s, the logistical process is influenced in part by the context of scientific research. Due to the research setting, the SOS-SAH intervention was often organized by research staff, who have more opportunities to remind patients to complete the questionnaire. The response percentage in clinical practice might therefore be lower. We nevertheless hope that patients will also be motivated to complete the SOS-SAH outside of a research setting, as the results are intended for use in their own care. We advise incorporating the questionnaire into the electronic patient record (EPR) in the future, thereby facilitating the use of the SOS-SAH.

We recommend that healthcare professionals discuss the results of the SOS-SAH with patients in order to interpret the PROM scores, for several reasons. First, not all of the patient complaints that are identified by the instrument are necessarily related to the aSAH. In addition, it is important to verify with patients whether the problems that have been identified are actually resulting in limitations and whether they wish to be treated for these problems. Importantly, the SOS-SAH may in some cases also underestimate or overlook the existence of problems experienced by patients. We therefore recommend that healthcare professionals also briefly check with their patients to verify that the smileys on the report accurately reflect the perceptions of the patients. Finally, it is important to realize that PROMs are no substitute for the dialogue between patients and healthcare professionals. This dialogue also provides information from non-verbal communication and the opportunity to explore complaints, in addition to meaningful and nuanced communication about relevant issues [14].

### Strengths and limitations of the study

One major strength of this study is the multi-method design. The qualitative study provides information on why the SOS-SAH is of benefit, and the quantitative study was intended to enrich this information with comparisons between data from usual-care and post-implementation patients. The multicenter design with four participating centers, increases external validity.

Our study is also subject to several limitations. First, the sample size in the implementation study was too small to allow any meaningful quantitative comparisons between the two groups. Second, the patients receiving usual care and those who were included in the implementation study were not completely comparable and additionally, the response percentage in the usual-care group is low, thereby possibly introducing bias. The patients who used the SOS-SAH had better WFNS scores and fewer symptoms. The group thus had an over-representation of patients with better outcomes after aSAH. Third, the number of participating healthcare professionals was too small to reach data saturation. Fourth, the interviews can be influenced by social desirability bias. That is that patients and participating healthcare professionals will be more inclined to give positive responses about the impact of the SOS-SAH. Last, this is an explorative study in which a limited number of healthcare professionals was involved, influencing external validity.

## Conclusion

The use of the SOS-SAH in daily clinical practice may help patients prepare better for their consultations and help to structure consultations between patients and healthcare professionals. We were not able to show an improvement of the quality of care experienced by patients, after implementation of the SOS-SAH. Both healthcare professionals and patients recommend continuing the use of the SOS-SAH in daily practice.

## Electronic supplementary material

Below is the link to the electronic supplementary material.

Supplementary Material 1

## Data Availability

The datasets generated and analysed during the current study are not publicly available due to the lack of specific patient consent to disclose the data, but are available from the corresponding author on reasonable request.
