# Supplementary material for: The SOS-SAH questionnaire in clinical practice: a multi-method evaluation study
Source: BMC Neurol. 2023 Jun 19;23:236. doi: 10.1186/s12883-023-03280-w (PMC10280905; doi:10.1186/s12883-023-03280-w)

**Supplemental material**

Supplemental material I: Additional information about the research team and interviews conducted to provide information for the COREQ checklist

Research team and reflexivity

EN conducted the interviews with patients and healthcare professionals based on the interview guides. EN is a PhD student and medical doctor following a residency in Neurology. EN had some training in qualitative research/interviewing, and had previously conducted interviews for qualitative research. Although she had treated patients after aSAH in the past, at the moment of the study, she was not a direct colleague of any of the healthcare professionals interviewed. Two of the participating healthcare professionals were former colleagues. EN developed the SOS-SAH, and the healthcare professionals were aware of this. At the start of the interviews, it was mentioned that the interviews were intended to gather their honest opinions about the questionnaire. The patients were not told that EN was the developer of the SOS-SAH. They were aware that the research was being conducted to evaluate their experiences with the SOS-SAH.

Study design

Sixteen patients were interviewed, each in a one-on-one setting. Six interviews were face-to-face. One of these interviews took place in the hospital, and the other five were conducted in the patient’s home. The other interviews were conducted by video call. The patient interviews lasted an average of 27 minutes, and the interviews with healthcare professionals lasted an average of 28 minutes.

Supplemental Table I. The SOS-SAH questionnaire.

| 1 | I have been able to bring to mind words that I wanted to use while talking to someone. | Not at all | A little bit | Somewhat | Quite a bit | Very much |
| --- | --- | --- | --- | --- | --- | --- |
| 2 | I have been able to focus my attention. | Not at all | A little bit | Somewhat | Quite a bit | Very much |
| 3 | I have been able to remember to do things, like take medicine or buy something I needed. | Not at all | A little bit | Somewhat | Quite a bit | Very much |
| 4 | I have been able to think clearly. | Not at all | A little bit | Somewhat | Quite a bit | Very much |
| 5 | I have been able to remember the name of a familiar object. | Not at all | A little bit | Somewhat | Quite a bit | Very much |
| 6 | I have been able to concentrate. | Not at all | A little bit | Somewhat | Quite a bit | Very much |
| 7 | I have been able to pay attention and keep track of what I am doing without extra effort. | Not at all | A little bit | Somewhat | Quite a bit | Very much |
| 8 | I have been able to learn new things easily, like telephone numbers or instructions. | Not at all | A little bit | Somewhat | Quite a bit | Very much |
| 9 | In a busy environment I find myself quickly bothered by excessive stimuli. | Not at all | A little bit | Somewhat | Quite a bit | Very much |
| 10 | I feel tense or 'wound up’. | Most of the time | A lot of the time | From time to time, occasionally | Not at all |  |
| 11 | I still enjoy the things I used to enjoy. | Definitely as much | Not quite so much | Only a little | Hardly at all |  |
| 12 | I get a sort of frightened feeling as if something awful is about to happen. | Very definitely and quite badly | Yes, but not too badly | A little, but it doesn't worry me | Not at all |  |
| 13 | I can laugh and see the funny side of things. | As much as I always could | Not quite so much now | Definitely not so much now | Not at all |  |
| 14 | Worrying thoughts go through my mind. | A great deal of the time | A lot of the time | From time to time but not too often | Only occasionally |  |
| 15 | I feel cheerful. | Not at all | Not often | Sometimes | Most of the time |  |
| 16 | I can sit at ease and feel relaxed. | Definitely | Usually | Not often | Not at all |  |
| 17 | I feel as if I am slowed down. | Nearly all the time | Very often | Sometimes | Not at all |  |
| 18 | I get a sort of frightened feeling like ‘butterflies’ in the stomach. | Not at all | Occasionally | Quite often | Very often |  |
| 19 | I have lost interest in my appearance. | Definitely | I don’t take so much care as I should | I may not take quite as much care | I take just as much care as ever |  |
| 20 | I feel restless as I have to be on the move. | Very much indeed | Quite a lot | Not very much | Not at all |  |
| 21 | I look forward with enjoyment to things. | As much as I ever did | Rather less than I used to | Definitely less than I used to | Hardly at all |  |
| 22 | I get sudden feelings of panic. | Very often indeed | Quite often | Not very often | Not at all |  |
| 23 | I can enjoy a good book or radio or TV programme. | Often | Sometimes | Not often | Very seldom |  |
| 24 | I feel fatigued. | Not at all | A little bit | Somewhat | Quite a bit | Very much |
| 25 | I have trouble starting things because I am tired. | Not at all | A little bit | Somewhat | Quite a bit | Very much |
| 26 | How run-down did you feel on average? | Not at all | A little bit | Somewhat | Quite a bit | Very much |
| 27 | How fatigued were you on average? | Not at all | A little bit | Somewhat | Quite a bit | Very much |
| 28 | I have trouble doing all of my regular leisure activities with others | Never | Rarely | Sometimes | Usually | Always |
| 29 | I have trouble doing all of the family activities that I want to do | Never | Rarely | Sometimes | Usually | Always |
| 30 | I have trouble doing all of my usual work (include work at home) | Never | Rarely | Sometimes | Usually | Always |
| 31 | I have trouble doing all of the activities with friends that I want to do | Never | Rarely | Sometimes | Usually | Always |
| 32 | I am a different person than I was before the (subarachnoid) hemorrhage. | Not at all | A little bit | Somewhat | Quite a bit | Very much |
| 33 | How difficult do you find it to hold a conversation? | Not at all | A little bit | Somewhat | Quite a bit | Very much |
| 34 | How difficult do you find it to follow a conversation? | Not at all | A little bit | Somewhat | Quite a bit | Very much |
| 35 | How much difficulty do you have with your sight? | Not at all | A little bit | Somewhat | Quite a bit | Very much |
| 36 | How much difficulty do you have with your sense of taste? | Not at all | A little bit | Somewhat | Quite a bit | Very much |
| 37 | How much difficulty do you have with your sense of smell? | Not at all | A little bit | Somewhat | Quite a bit | Very much |
| 38 | How much difficulty do you have with your hearing? | Not at all | A little bit | Somewhat | Quite a bit | Very much |
| 39 | How bothered are you by headaches? | Not at all | A little bit | Somewhat | Quite a bit | Very much |
| 40 | Has the (subarachnoid) hemorrhage affected your sex life? | Not at all | A little bit | Somewhat | Quite a bit | Very much |
|  | **Proxy questions for family members** |  |  |  |  |  |
| 41 | My family member is a different person than he/she was before the (subarachnoid) hemorrhage. | Not at all | A little bit | Somewhat | Quite a bit | Very much |
| 42 | My family member has been able to bring to mind words that he/she wanted to use while talking to someone. | Not at all | A little bit | Somewhat | Quite a bit | Very much |
| 43 | My family member has been able to focus his/her attention. | Not at all | A little bit | Somewhat | Quite a bit | Very much |
| 44 | My family member has been able to remember to do things, like take medicine or buy something he/she needed. | Not at all | A little bit | Somewhat | Quite a bit | Very much |
| 45 | My family member has been able to think clearly. | Not at all | A little bit | Somewhat | Quite a bit | Very much |
| 46 | My family member has been able to remember the name of a familiar object. | Not at all | A little bit | Somewhat | Quite a bit | Very much |
| 47 | My family member has been able to concentrate. | Not at all | A little bit | Somewhat | Quite a bit | Very much |
| 48 | My family member has been able to pay attention and keep track of what he/she is doing without extra effort. | Not at all | A little bit | Somewhat | Quite a bit | Very much |
| 49 | My family member has been able to learn new things easily, like telephone numbers or instructions. | Not at all | A little bit | Somewhat | Quite a bit | Very much |

SOS-SAH: Questionnaire for Screening on Symptoms in aneurysmal Subarachnoid Hemorrhage

Supplemental Table 2. The adapted questions for patients after aSAH from the CQI survey.

|  |  |
| --- | --- |
| 1 | How often have you had a subarachnoid hemorrhage?  ❑ Once ❑ Twice ❑ Three times ❑ More than three times |
| 2 | When did you have the (most recent) subarachnoid hemorrhage?  Month ……….  Year……………. |
| 3 | Did you receive any therapy or rehabilitation therapy after the subarachnoid hemorrhage?  ❑ No 🡪 continue to Question 5 ❑ Yes |
| 4 | Where did you receive this rehabilitation therapy? (multiple answers possible)  ❑ Hospital  ❑ Nursing home during admission ❑ Nursing home (by appointment) ❑ Rehabilitation center during admission ❑ Rehabilitation center (by appointment) ❑ Health center in the neighborhood ❑ At home ❑ Other: |
| 5 | Have you had follow-up appointments because of your subarachnoid hemorrhage in the past 12 months?  ❑ No 🡪 continue to Question 11 ❑ Yes, in a hospital ❑ Yes, in a nursing home ❑ Yes, in a rehabilitation clinic ❑ Yes, with the general practitioner ❑ Yes, at home ❑ Yes, other: |
| 6 | With which healthcare professionals did you have these follow-up appointments? (multiple answers possible) ❑ Nurse or nurse practitioner specialized in subarachnoid hemorrhage  ❑ Neurosurgeon ❑ Neurologist ❑ Interventional radiologist ❑ Geriatric specialist ❑ Rehabilitation specialist ❑ General practitioner ❑ Primary care assistant practitioner  ❑ Other: |
| 7 | With which healthcare professionals have you had the most contact for follow-up? (only one answer possible; please indicate that healthcare professional with whom you have had most appointments)  ❑ Nurse or nurse practitioner specialized in subarachnoid hemorrhage  ❑ Neurosurgeon ❑ Neurologist ❑ Interventional radiologist ❑ Geriatric specialist ❑ Rehabilitation specialist ❑ General practitioner ❑ Primary care assistant practitioner  ❑ Other: |
| 8 | Was your need for more or different care discussed during the follow-up appointments?  ❑ Not at all ❑ A little ❑ To a great extent ❑ Yes, completely |
| 9 | Was attention paid to problems you have encountered in carrying out daily activities (e.g., dressing and cooking)?  ❑ Not at all ❑ A little ❑ To a great extent ❑ Yes, completely |
| 10 | Was attention paid to your emotions (e.g., how to deal with the consequences of the SAH or fear of another SAH)?  ❑ Not at all ❑ A little ❑ To a great extent ❑ Yes, completely |
| 11 | Have you had contact with multiple healthcare professionals in the past 12 months because of your SAH?  ❑ No 🡪 continue to Question 16 ❑ Yes, multiple healthcare professionals in the hospital ❑ Yes, multiple healthcare professionals inside and outside the hospital |
| 12 | Were the various healthcare professionals aware of your medical condition?  ❑ Not at all ❑ A little ❑ To a great extent ❑ Yes, completely ❑ I don’t know (anymore) |
| 13 | Was there good cooperation between the various healthcare professionals?  ❑ Not at all ❑ A little ❑ To a great extent ❑ Yes, completely ❑ I don’t know (anymore) |
| 14 | Did the care received from the various healthcare professionals correspond with each other?  ❑ Not at all ❑ A little ❑ To a great extent ❑ Yes, completely ❑ I don’t know (anymore) |
| 15 | Did the information received from the different healthcare professionals correspond with each other?  ❑ Not at all ❑ A little ❑ To a great extent ❑ Yes, completely ❑ I don’t know (anymore) |
| 16 | Did you receive information about patient associations or representative associations?  ❑ No ❑ Yes |
| 17 | Did you receive information about the possibilities of future support or guidance (e.g., about contacts with social work, a psychologist, or specialized nurse)?  ❑ No ❑ Yes |
| 18 | Did you receive information about the possibilities of support for the person/people close to you (e.g., partner, relatives)?  ❑ No ❑ Yes ❑ Not applicable, no partner or relatives involved |
| 19 | Did the recommendations of your healthcare professionals correspond to your wishes and possibilities?  ❑ Not at all ❑ A little ❑ To a great extent ❑ Yes, completely |
| 20 | Did you receive assistance in finding your way within the healthcare sector?  ❑ Not at all ❑ A little ❑ To a great extent ❑ Yes, completely ❑ Not applicable, not necessary |
| 21 | Did you receive help in gaining access to medical devices, medical facilities, or home-based care?  ❑ Not at all ❑ A little ❑ To a great extent ❑ Yes, completely ❑ Not applicable, not necessary |
| 22 | Have you had a designated contact in the past 12 months for questions about your subarachnoid hemorrhage?  ❑ No ❑ Yes |
| 23 | Who was this designated contact?  ❑ Specialized nurse in subarachnoid hemorrhage or nurse practitioner ❑ Neurosurgeon ❑ Neurologist ❑ Interventional radiologist ❑ Social worker ❑ General practitioner ❑ Primary care assistant practitioner  ❑ Social caregiver  ❑ Other: |
| 24 | What is your age? |
| 25 | Are you a man or a woman?  ❑ Man ❑ Woman |
| 26 | What is the highest degree or level of education you have completed (with a diploma or certificate)?  ❑ No degree (did not finish primary education) ❑ Basic preparatory secondary vocational education ❑ Preparatory secondary vocational education ❑ Intermediate general secondary education ❑ Senior secondary vocational training ❑ Senior secondary general education/pre-university education ❑ Higher professional education (Bachelor’s degree)  ❑ Academic higher education (Master’s degree) ❑ Other: |
| 27 | How would you rate your health status in general?  ❑ Excellent ❑ Very good ❑ Good ❑ Fair ❑ Poor |
| 28 | How would you rate your current health status, compared to your health status immediately after discharge from the hospital? My health is now...  ❑ Much better ❑ Somewhat better ❑ About the same ❑ Somewhat worse ❑ Much worse |
| 29 | Did the extent of recovery meet your expectations?  ❑ Not at all ❑ A little ❑ To a great extent ❑ Yes, completely |
| 30 | What is your country of birth? |
| 31 | Please indicate the health changes you encountered after your subarachnoid hemorrhage and whether you discussed these changes with your healthcare professional?  Changes in or with:   - Moving one or both arms   ❑ Yes ❑ No  If yes, did you discuss this complaint during your follow-up appointment in the hospital?  ❑ Yes ❑ No   - Moving one or both legs   ❑ Yes ❑ No  If yes, did you discuss this complaint during your follow-up appointment in the hospital?  ❑ Yes ❑ No   - Balance (for example, dizziness)   ❑ Yes ❑ No  If yes, did you discuss this complaint during your follow-up appointment in the hospital?  ❑ Yes ❑ No   - Energy (for example fatigue)   ❑ Yes ❑ No  If yes, did you discuss this complaint during your follow-up appointment in the hospital?  ❑ Yes ❑ No   - Swallowing   ❑ Yes ❑ No  If yes, did you discuss this complaint during your follow-up appointment in the hospital?  ❑ Yes ❑ No   - Talking   ❑ Yes ❑ No  If yes, did you discuss this complaint during your follow-up appointment in the hospital?  ❑ Yes ❑ No   - Seeing   ❑ Yes ❑ No  If yes, did you discuss this complaint during your follow-up appointment in the hospital?  ❑ Yes ❑ No   - Hearing   ❑ Yes ❑ No  If yes, did you discuss this complaint during your follow-up appointment in the hospital?  ❑ Yes ❑ No   - Tasting   ❑ Yes ❑ No  If yes, did you discuss this complaint during your follow-up appointment in the hospital?  ❑ Yes ❑ No   - Smelling   ❑ Yes ❑ No  If yes, did you discuss this complaint during your follow-up appointment in the hospital?  ❑ Yes ❑ No   - Thinking (for example, concentration, remembering things, orientation in place and time)   ❑ Yes ❑ No  If yes, did you discuss this complaint during your follow-up appointment in the hospital?  ❑ Yes ❑ No   - Emotions (for example, crying, depressive feelings)   ❑ Yes ❑ No  If yes, did you discuss this complaint during your follow-up appointment in the hospital?  ❑ Yes ❑ No   - Behavior (for example, anger, impatience)   ❑ Yes ❑ No  If yes, did you discuss this complaint during your follow-up appointment in the hospital?  ❑ Yes ❑ No   - Initiative and interests   ❑ Yes ❑ No  If yes, did you discuss this complaint during your follow-up appointment in the hospital?  ❑ Yes ❑ No   - Working   ❑ Yes ❑ No  If yes, did you discuss this complaint during your follow-up appointment in the hospital?  ❑ Yes ❑ No   - Activities with family   ❑ Yes ❑ No  If yes, did you discuss this complaint during your follow-up appointment in the hospital?  ❑ Yes ❑ No   - Hobbies   ❑ Yes ❑ No  If yes, did you discuss this complaint during your follow-up appointment in the hospital?  ❑ Yes ❑ No   - Headache   ❑ Yes ❑ No  If yes, did you discuss this complaint during your follow-up appointment in the hospital?  ❑ Yes ❑ No   - Urination and/or defecation (incontinence)   ❑ Yes ❑ No  If yes, did you discuss this complaint during your follow-up appointment in the hospital?  ❑ Yes ❑ No   - Sexuality   ❑ Yes ❑ No  If yes, did you discuss this complaint during your follow-up appointment in the hospital?  ❑ Yes ❑ No   - Other:   ❑ Yes ❑ No  If yes, did you discuss this complaint during your follow-up appointment in the hospital?  ❑ Yes ❑ No |

The scales that were used are composed of the following questions:
1) Follow-up care in the outpatient clinic: Questions 8, 9, and 10; 2) Collaboration between healthcare professionals: Questions 12, 13, and 14; 3) Information about support options: Questions 16, 17, 18, and 22; 4) Support in gaining access to healthcare or medical devices: Questions 20 and 21.

Supplemental Table 3. Overview of symptoms discussed and not discussed for the usual-care group and the post-implementation group who used the SOS-SAH.

| **Difficulties with:** |  |  |  |  |
| --- | --- | --- | --- | --- |
|  |  | **Usual care**  **(n = 79)** | **Post-implementation (n = 34)** |  |
| Moving one or both arms | Yes and discussed | 9 (12.0%) | 5 (14.7%) |  |
|  | Yes and not discussed | 1 (1.3%) | 1 (2.9%) |  |
| Moving one or both legs | Yes and discussed | 11 (14.5%) | 7 (20.6%) |  |
|  | Yes and not discussed | 3 (3.9%) | 1 (2.9%) |  |
| Balance | Yes and discussed | 22 (29.7%) | 6 (18.2%) |  |
|  | Yes and not discussed | 7 (9.5%) | 2 (6.1%) |  |
| Energy | Yes and discussed | 52 (68.4%) | 21 (61.8%) |  |
|  | Yes and not discussed | 6 (7.9%) | 3 (8.8%) |  |
| Swallowing | Yes and discussed | 5 (6.6%) | 0 |  |
|  | Yes and not discussed | 4 (5.3%) | 0 |  |
| Talking | Yes and discussed | 12 (15.8%) | 4 (12.1%) |  |
|  | Yes and not discussed | 4 (5.3%) | 0 |  |
| Seeing | Yes and discussed | 21 (27.6%) | 2 (5.9%) |  |
|  | Yes and not discussed | 3 (3.9%) | 1 (2.9%) |  |
| Hearing | Yes and discussed | 3 (4.0%) | 4 (11.8%) |  |
|  | Yes and not discussed | 2 (2.7%) | 0 |  |
| Tasting | Yes and discussed | 4 (5.3%) | 2 (5.9%) |  |
|  | Yes and not discussed | 4 (5.3%) | 0 |  |
| Smelling | Yes and discussed | 1 (1.3%) | 0 |  |
|  | Yes and not discussed | 1 (1.3%) | 1 (2.9%) |  |
| Thinking | Yes and discussed | 40 (51.9%) | 20 (58.8%) |  |
|  | Yes and not discussed | 8 (10.4%) | 3 (8.8%) |  |
| Mood | Yes and discussed | 27 (36.5%) | 9 (26.5%) |  |
|  | Yes and not discussed | 8 (10.8%) | 3 (8.8%) |  |
| Behavior | Yes and discussed | 23 (30.3%) | 3 (8.8%) |  |
|  | Yes and not discussed | 8 (10.5%) | 7 (20.6%) |  |
| Taking initiative or having interests | Yes and discussed | 15 (19.7%) | 4 (11.8%) |  |
|  | Yes and not discussed | 12 (15.8%) | 4 (11.8%) |  |
| Working | Yes and discussed | 29 (39.7%) | 12 (36.4%) |  |
|  | Yes and not discussed | 12 (16.4%) | 4 (12.1%) |  |
| Activities with family | Yes and discussed | 15 (20.0%) | 4 (12.1%) |  |
|  | Yes and not discussed | 7 (9.3%) | 3 (9.1%) |  |
| Hobbies | Yes and discussed | 16 (21.3%) | 4 (12.5%) |  |
|  | Yes and not discussed | 11 (14.7%) | 2 (6.3%) |  |
| Headache | Yes and discussed | 19 (25.3%) | 9 (29.0%) |  |
|  | Yes and not discussed | 5 (6.7%) | 1 (3.2%) |  |
| Urination and/or defecation | Yes and discussed | 12 (16.0%) | 2 (6.1%) |  |
|  | Yes and not discussed | 5 (6.7%) | 1 (3.0%) |  |
| Sexuality | Yes and discussed | 2 (2.7%) | 0 |  |
|  | Yes and not discussed | 9 (12.2%) | 1 (3.0%) |  |
| Other symptoms | Yes and discussed | 4 (33.3%) | 1 (16.7%) |  |
|  | Yes and not discussed | 1 (8.3%) | 0 |  |
| Number of symptoms per patient |  | 6.1 | 5.2 |  |
| Total score discussed |  | 342 | 119 |  |
| Total score not discussed |  | 121 | 37 |  |
| Ratio of not discussed to discussed |  | 0.35 | 0.31 |  |

Supplemntal Figure 1. The SOS-SAH summary report.


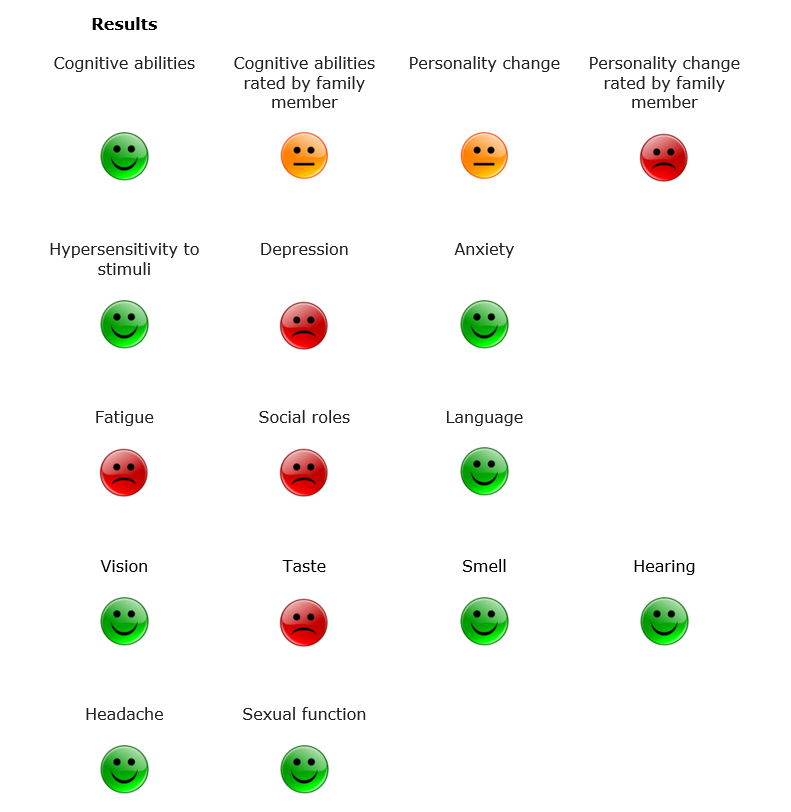

Supplement: Supplementary file 1 — Supplementary Material 1 [file 12883_2023_3280_MOESM1_ESM.docx]
